# Right ventricular contraction patterns in healthy children using three-dimensional echocardiography

**DOI:** 10.3389/fcvm.2023.1141027

**Published:** 2023-08-03

**Authors:** Christopher Valle, Adrienn Ujvari, Eleni Elia, Minmin Lu, Naomi Gauthier, David Hoganson, Gerald Marx, Andrew J. Powell, Alessandra Ferraro, Bálint Lakatos, Zoltán Tősér, Béla Merkely, Attila Kovacs, David M. Harrild

**Affiliations:** ^1^ Department of Cardiology, Boston Children’s Hospital, Boston, MA, United States; ^2^ Department of Pediatrics, Harvard Medical School, Boston, MA, United States; ^3^ Heart and Vascular Center, Semmelweis University, Budapest, Hungary; ^4^ School of Engineering, Computing and Mathematics, Oxford Brookes University, Oxford, United Kingdom; ^5^ Department of Cardiac Surgery, Boston Children’s Hospital, Boston, MA, United States; ^6^ Argus Cognitive, Inc., Lebanon, NH, United States

**Keywords:** right ventricle, three dimensional echocardiography, ventricular mechanics, myocardial deformation, pediatric cardiology

## Abstract

**Background:**

The right ventricle (RV) has complex geometry and function, with motion along three separate axes—longitudinal, radial, and anteroposterior. Quantitative assessment of RV function by two-dimension echocardiography (2DE) has been limited as a consequence of this complexity, whereas newer three dimensional (3D) analysis offers the potential for more comprehensive assessment of the contributors to RV function. The aims of this study were to quantify the longitudinal, radial and anteroposterior components of global RV function using 3D echocardiography in a cohort of healthy children and to examine maturational changes in these parameters.

**Methods:**

Three-dimensional contours of the RV were generated from a cohort of healthy pediatric patients with structurally normal hearts at two centers. Traditional 2D and 3D echo characteristics were recorded. Using offline analysis of 3D datasets, RV motion was decomposed into three components, and ejection fractions (EF) were calculated (longitudinal-LEF; radial-REF; and anteroposterior-AEF). The individual decomposed EF values were indexed against the global RVEF. Strain values were calculated as well.

**Results:**

Data from 166 subjects were included in the analysis; median age was 13.5 years (range 0 to 17.4 years). Overall, AEF was greater than REF and LEF (29.2 ± 6.2% vs. 25.1 ± 7.2% and 25.7 ± 6.0%, respectively; *p* < 0.001). This remained true when indexed to overall EF (49.8 ± 8.7% vs. 43.3 ± 11.6% and 44.4 ± 10%, respectively; *p* < 0.001). Age-related differences were present for global RVEF, REF, and all components of RV strain.

**Conclusions:**

In healthy children, anteroposterior shortening is the dominant component of RV contraction. Evaluation of 3D parameters of the RV in children is feasible and enhances the overall understanding of RV function, which may allow improvements in recognition of dysfunction and assessment of treatment effects in the future.

## Introduction

Accurate assessment of RV morphology and function are of critical importance in cardiovascular disease in children and adolescents, particularly in complex congenital heart disease involving systemic RV physiology (1–3), obstructive right-sided heart disease (4, 5) and pulmonary hypertension (6, 7). However, compared to the established tools and techniques developed for assessment of the LV, evaluation of the complex anatomy and contraction of the RV by two-dimensional (2D) echocardiography remains significantly limited in clinical practice. Recently, however, three-dimensional echocardiography (3DE) has been shown to be able to produce excellent quantification of RV volumes and has been validated against gold-standard modalities such as cardiac magnetic resonance imaging, even in children with complex congenital heart disease (8–10).

The pattern of right ventricular (RV) contraction is dictated by its complex structure on both gross and microscopic levels. In the normal RV, two myofiber layers are present: a subendocardial layer that consists primarily of longitudinally aligned fibers and a subepicardial layer that consists primarily of circumferentially oriented fibers (11–14). This arrangement results in three primary contributors to RV ejection: (1) traction of the tricuspid annulus toward the apex leading to longitudinal shortening; (2) a “bellows”-like inward movement of the RV free wall leading to radial shortening; and (3) traction of the RV free wall associated with left ventricular (LV) deformation, leading to anteroposterior shortening (15, 16).

Accordingly, the aim of this study was to use a novel 3DE-based analysis technique to develop foundational data describing the relative contributions of longitudinal, radial, and anteroposterior motion components of global RV function in a cohort of healthy children with structurally normal hearts. Specifically, we sought to examine differences in the relative contributions of the 3 components of ejection fraction in children and to look for changes in the contribution of these components as a function of age.

## Methods

Healthy children age <18 years were included from two centers: Boston Children's Hospital, Boston, MA, USA; and the Heart and Vascular Center of the Semmelweis University, Budapest, Hungary. Subjects at the Boston site were identified retrospectively from an existing database of 3DE images with accompanying clinical and demographic information. Patients in this database had presented to the outpatient clinic between 2014 and 2020 for evaluation of a common cardiac condition (most frequently murmur, chest pain, syncope, or family history of cardiac condition), were judged to have a structurally and functionally normal heart, and were discharged from further follow-up. Exclusion criteria included structural abnormalities other than patent foramen ovale or trivial branch pulmonary stenosis (maximum instantaneous gradient < 15 mmHg within the first two years of life); arrhythmia (other than rare atrial or ventricular premature beats) including sinus bradycardia or tachycardia (heart rate z-score < −2 or >  + 2 for age), acquired heart disease (cardiomyopathy, chemotherapy exposure, and Kawasaki disease), or co-morbidities with a potential impact on ventricular size and function (i.e., hypertension, renal failure, anemia, history of prematurity, chronic lung disease, pulmonary hypertension, obstructive sleep apnea, and connective tissue disorder).

Healthy volunteers at the Semmelweis site were recruited from local schools; no individuals were identified subsequently with significant cardiac abnormalities revealed by echocardiography, electrocardiography, blood pressure measurement, or review of medical history**.** Study protocols were approved by both centers' institutional review boards. Given the retrospective nature of recruitment at Boston Children's Hospital, informed consent was waived at that site. At Semmelweiss University, families of all participants provided written informed consent to participate in the study.

Blood pressure, height, and weight were recorded for all subjects. Body surface area (BSA) was calculated using the Mosteller formula (17).

### 2D and 3d echocardiography

Echocardiographic acquisitions were performed using the Philips (IE33 and Epiq, Philips, Cambridge, MA) and GE (E95, GE Healthcare, Horten, Norway) ultrasound systems, in accordance with the American Society of Echocardiography (ASE) standards for performing a pediatric echocardiogram (18). Parameters recorded from the 2D echo images included tricuspid annular plane systolic excursion (TAPSE), RV length, fractional area change (FAC), and qualitative assessment of the degree of tricuspid regurgitation. LV volumes were calculated from the 5/6 × area × length formula and presented in raw fashion, as well as being indexed to BSA.

In addition to the standard 2D echocardiographic protocol, electrocardiographically gated full-volume 3D data sets reconstructed from four or six cardiac cycles optimized for RV views were obtained for offline analysis. At the Semmelweiss site, images were obtained from the apical window using the 4Vc-D transducer (GE Healthcare, Horten, Norway). At the Boston site, images were obtained from the apical or subcostal window using the X5 probe (Philips, Cambridge, MA) in a patient-specific fashion (i.e., the window providing better image quality was used). Image quality was verified at the bedside to minimize stitching and dropout artifacts of the 3D data; breath-holding manuevers were used as appropriate for the developmental age of the child.

3D datasets were analyzed off-line using dedicated software (4D RV-Function; TomTec Imaging, Unterschleissheim, Germany). The algorithm detects the endocardial surface of the RV and, following manual correction, traces its motion throughout the cardiac cycle. End-diastolic volume, end-systolic volume, stroke volume, and free wall longitudinal strain were recorded.

### Analysis of 3D components of right ventricular contraction

**Figure 1:**
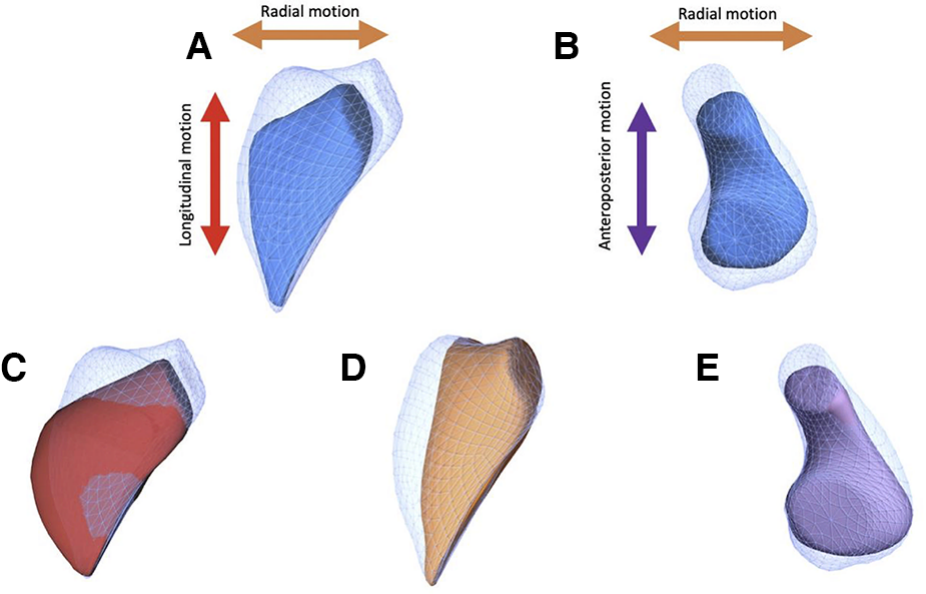
The three different components of right ventricular contraction from a representative subject. In the figure the global motion of the right ventricle is shown from anterior (**A**) and superior (**B**) views (blue mesh, RV end-diastolic volume; blue surface, RV end-systolic volume). RV end-systolic meshes can be generated by “locking” the RV motion in two directions, permitting motion in only a single axis and thus revealing the impact of decomposed contraction components. Thus, the change in ventricular volume attributable to shortening along the longitudinal (**C**, red surface), radial (**D**, orange surface) and anteroposterior (**E**, purple surface) can be separately quantified.

The 3D RV deformation analysis used has been previously described in detail (19, 20). Briefly, the constructed 3D meshes were exported from TomTec 4D RV Function. Then, using the ReVISION software (Argus Cognitive, Lebanon, New Hampshire), the workflow consists of the following steps: (1) standardization of the 3D mesh orientation, (2) movement decomposition, and (3) calculation of volumes and strain values. Orientation adjustment was performed by a rule-based, automated method to define longitudinal, radial and anteroposterior directions as described in tetail elsewere (20). Motion decomposition along the aforementioned, orthogonal, anatomically relevant axes is performed in a vertex-based manner. By the decomposition of the model's motion along the three anatomically relevant orthogonal axes, the volume change of the RV attributable to each specific direction was determined (Figure 1).

Therefore, we measured component EF values (longitudinal EF—LEF, radial EF—REF, and anteroposterior EF—AEF). These raw decomposed EF values were then indexed to global RVEF (i.e., indexed AEF = AEF/global RVEF) to genertate the longitudinal EF index (LEFi), radial EF index (REFi), and anteroposterior EF index (AEFi). These measures quantify the relative contribution of the given direction to global RV performance. Note that the absolute volume change of the chamber is generated by the aggregated contribution of the three motion components. This composition is not additive, and consequentially, the sum of the decomposed volume changes is not equal to the global volume change; in other words, the relative contribution of the motion components do not add up to 100%. Global and decomposed volumes are calculated using the signed tetrahedron method (19).

To assess myocardial deformation, predefined longitudinally and circumferentially-oriented contours were used, and 3D global longitudinal strain (GLS) and global circumferential strain (GCS) were computed as previously described (20). 3D global area strain (GAS) was also calculated by the relative change of the endocardial surface between end-diastole and end-systole.

### Statistical analysis

Continuous data were presented as mean ± standard deviation (SD) or median and interquartile range. Categorical data were presented as counts and percentages (% of total population). Outcomes were summarized according to age groups representing different categories of patient body size: *Infants*: <1 year, *Toddlers* >1–5 years, *School-Aged*: >5–10 years, *(Pre)Teens* > 10–18 years. One-way ANOVA or the Kruskal Wallis H test was performed to compare the distribution of parameters by age group as appropriate. Wilcoxon signed-rank test was used to assess for differences in the contribution of LEF, REF, and AEF within each pre-specified age group, with Bonferroni correction applied (i.e., level of statistical significance set at *p *< 0.017). In order to assess the impact of patient sex on the ejection fraction parameters, a general linear model was used to compare EF means by sex with adjustment for age to produce least-squares means.

To assess intercenter reproducibility, one operator from the Boston site and one from the Semmelweis site each reviewed a subset of 30 patients, blinded to the other's results. The strength of agreement was assessed by intraclass correlation coefficients (ICC) along with the Bland Altman plot.

Data analyses were performed with SAS software (version 9.4, SAS Institute Inc., Cary, North Carolina) and R 4.1.2 (2021 The R Foundation for Statistical Computing Platform). *P* values <0.05 were used to indicate statistical significance.

## Results

The study population included 166 subjects (Boston = 76; Semmelweis = 90). Demographic and clinical characteristics of the study population are summarized in Table 1. The median age of subjects was 13.8 years (IQR 8.6 to 15.3), with a skewed distribution towards the oldest age group (as a consequence of the recruitment strategy at the Semmelweis site). The population was majority male (*n* = 131, 79%), driven by a male-predominant population recruited at the Semmelweiss site (*n* = 81, 90%).

**Table 1:** Patient characteristics.

	All (*n* = 166)	Infants (*n* = 13)	Toddlers (*n* = 11)	School-Aged (*n* = 21)	(Pre)Teens (*n* = 121)	*p*-value
Age, year	13.8 (8.6, 15.3)	0.1 (0.05, 0.1)	3.6 (3.3, 4.1)	6.3 (5.2, 7.9)	14.4 (13.6,15.7)	<0.001
Female [*n* (%)]	35 (21%)	7 (54%)	5 (45%)	7 (33%)	16 (13%)	
Height, m	1.49 ± 0.38	0.53 ± 0.07	1.00 ± 0.08	1.21 ± 0.11	1.69 ± 0.14	<0.001
Weight, kg	47.7 ± 23.8	4.2 ± 2.0	16.6 ± 3.2	23.3 ± 5.9	59.4 ± 15.3	<0.001
BMI, kg/m^2^	19.1 ± 3.5	14.3 ± 2.0	16.3 ± 1.4	15.5 ± 1.5	20.5 ± 3.0	<0.001
BSA, m^2^	1.39 ± 0.54	0.25 ± 0.07	0.68 ± 0.09	0.88 ± 0.14	1.66 ± 0.29	<0.001
SBP, mm Hg	117 ± 19	92 ± 14	100 ± 10	100 ± 10	125 ± 16	<0.001
DBP, mm Hg	65 ± 12	53 ± 11	53 ± 6	56 ± 8	68 ± 10	<0.001
HR, beats/min	80 ± 20	130 ± 13	89 ± 12	84 ± 14	73 ± 12	<0.001

Continuous data are expressed as mean ±SD, with the exception of age which are presented as median (IQR).

BMI, body mass index; BSA, body surface area; DBP, diastolic blood pressure; HR, heart rate; SBP, systolic blood pressure.

Conventional echocardiographic measures of RV and LV function are presented in Table 2. Tricuspid annular plane systolic excursion (TAPSE) increased significantly with age. Most subjects had either no (92, 53%) or trivial (72, 42%) tricuspid regurgitation. There were no differences between groups in terms of RV FAC. Age-related variation in 2D free wall longitudinal strain was present, with the largest absolute values seen in the toddler and school-aged groups.

**Table 2:** Conventional echocardiographic characteristics.

	All (*n* = 166)	Infants (*n* = 13)	Toddlers (*n* = 11)	School-Aged (*n* = 21)	(Pre)Teens (*n* = 121)	*p*-value
TAPSE, mm	21.8 (16.9, 26.1)	6.2 (4.5, 9.0)	14.3 (12.0, 19.7)	17.1 (15.3, 19.6)	23.7 (20.9, 27.8)	<.001
RV FAC, %	48.8 (45.3, 52.4)	46.5 (42.9, 51.2)	51.3 (44.7, 54.2)	50.7 (48.7, 54.2)	48.5 (45.3, 52.2)	0.161
RV 2D FWLS, %	−30.4 (−33.7, −26.2)	−29.8 (−31.8, −23.2)	−34.8 (−39.0, −28.4)	−32.1 (−36.6, −30.9)	−29.4 (−33.0, −26.2)	0.016
2D LVEDV, ml	113.9 (74.6, 145.6)	9.4 (7.7, 11.1)	48.6 (39.0, 55.9)	62.8 (55.7, 74.6)	135.0 (110.9, 154.6)	<.001
2D LVEDVi, ml/m^2^	77.2 (67.1, 85.8)	42.7 (35.0, 43.5)	70.8 (60.9, 75.5)	70.4 (66.8, 79.3)	79.8 (73.0, 88.9)	<.001
2D LVESV, ml	45.2 (26.3, 60.9)	3.7 (3.0, 3.9)	17.3 (12.5, 18.2)	21.3 (20.1, 26.3)	54.8 (42.5, 66.1)	<.001
2D LVESVi, ml/m^2^	30.1 (24.9, 35.4)	14.6 (12.7, 16.5)	23.4 (21.1, 25.8)	25.3 (23.4, 26.8)	32.7 (28.1, 36.7)	<.001
LV EF, %	60.4 (57.0, 63.9)	61.5 (59.0, 64.3)	65.9 (64.0, 67.9)	64.0 (62.6, 67.0)	58.9 (56.3, 62.0)	<.001

Data are expressed as median (Q1, Q3).

2D, two-dimensional; EF, ejection fraction; FAC, fractional area change; FWLS, free wall longitudinal strain; LVEDVi, indexed left ventricular end-diastolic volume; LVESVi, indexed left ventricular end-systolic volume; RV, right ventricular; TAPSE, tricuspid annulus plane systolic excursion.

Table 3 presents 3D RV volumes and contraction patterns. RV volumes, global RVEF, REF and REFi, longitudinal and circumferential 3D strain parameters significantly differ by age group. Age-related differences were present for global RVEF, REF and REFi. Additionally, age-related differences were seen for all components of RV strain. Supplementary Table S1 presents sex-specific age-adjusted mean values for the ejection fraction parameters; no differences were identified between male and female subgroups.

**Table 3:** Three-dimensional echocardiographic analysis of right ventricular size and ejection fraction components.

	All (*n* = 166)	Infants (*n* = 13)	Toddlers (*n* = 11)	School-aged (*n* = 21)	(Pre)Teens (*n* = 121)	*p*-value
3D RVEDV, ml	115.3 (66.0, 149.6)	9.4 (8.0, 10.0)	43.5 (37.5, 47.7)	56.3 (52.4, 64.3)	133.4 (107.2, 157.0)	**<** **.** **001**
3D RVEDVi, ml/m^2^	74.8 (64.4, 87.3)	40.7 (38.0, 43.1)	64.4 (59.4, 67.9)	66.4 (59.9, 73.8)	80.1 (71.0, 89.9)	**<** **.** **001**
3D RVESV, ml	47.1 (25.6, 63.3)	3.9 (3.3, 4.9)	16.4 (15.2, 19.5)	22.4 (18.4, 25.6)	56.8 (43.0, 70.3)	**<** **.** **001**
3D RVESVi, ml/m^2^	31.6 (24.6, 37.4)	16.0 (14.9, 18.8)	24.6 (22.2, 28.1)	25.8 (23.4, 27.4)	33.8 (28.8, 39.4)	**<** **.** **001**
RV EF, %	58.1 (54.6, 61.4)	55.1 (52.7, 61.1)	59.3 (55.0, 65.0)	62.1 (58.4, 64.6)	57.3 (54.3, 61.0)	**0** **.** **008**
LEF, %	25.8 (22.1, 29.8)	23.0 (19.3, 27.2)	27.6 (22.7, 35.8)	28.0 (23.4, 29.8)	25.8 (21.3, 29.7)	0.345
REF, %	25.3 (20.3, 30.6)	28.7 (22.6, 32.2)	26.8 (22.3, 31.7)	28.2 (25.7, 32.2)	24.1 (19.5, 29.7)	**0** **.** **020**
AEF, %	28.8 (24.8, 32.9)	27.6 (24.1, 33.8)	31.2 (27.2, 37.5)	32.6 (25.1, 36.4)	28.3 (24.7, 32.2)	0.109
LEFi, %	43.8 (39.1, 50.2)	43.0 (33.7, 43.7)	43.7 (40.8, 57.5)	43.7 (42.1, 51.1)	44.9 (38.2, 49.9)	0.436
REFi, %	43.7 (35.3, 51.3)	48.2 (42.8, 53.6)	45.8 (33.8, 59.2)	48.0 (43.4, 50.8)	42.4 (34.0, 51.1)	0.055
AEFi, %	50.7 (43.1, 55.6)	52.2 (41.5, 54.4)	57.2 (46.6, 60.9)	53.0 (42.1, 58.2)	50.5 (43.5, 54.0)	0.353
3D GAS, %	−40.3 (−43.7, −37.3)	−37.6 (−43.8, −34.4)	−43.8 (−44.8, −37.7)	−43.1 (−45.0, −39.0)	−40.1 (−42.7, −37.0)	**0** **.** **046**
3D GLS, %	−22.9 (−25.8, −20.5)	−18.9 (−23.7, −17.5)	−25.9 (−26.8, −22.7)	−24.5 (−25.8, −21.1)	−22.8 (−25.6, −20.9)	**0** **.** **029**
3D GCS, %	−23.6 (−26.6, −19.9)	−24.9 (−26.9, −20.9)	−25.6 (−27.0, −17.1)	−26.6 (−29.1, −22.5)	−22.7 (−25.9, −19.7)	**0** **.** **031**

Data are expressed as median (Q1, Q3).

3D, three-dimensional; AEF, anteroposterior ejection fraction; EF, ejection fraction; GAS, global area strain; GCS, global circumferential strain; GLS, global longitudinal strain; LEF, longitudinal ejection fraction; REF, radial ejection fraction; RVEDVi, indexed right ventricular end-diastolic volume; RVESVi, indexed right ventricular end-systolic volume.

Bolded values are statistically significant (*p* < 0.05).

**Figure 2:**
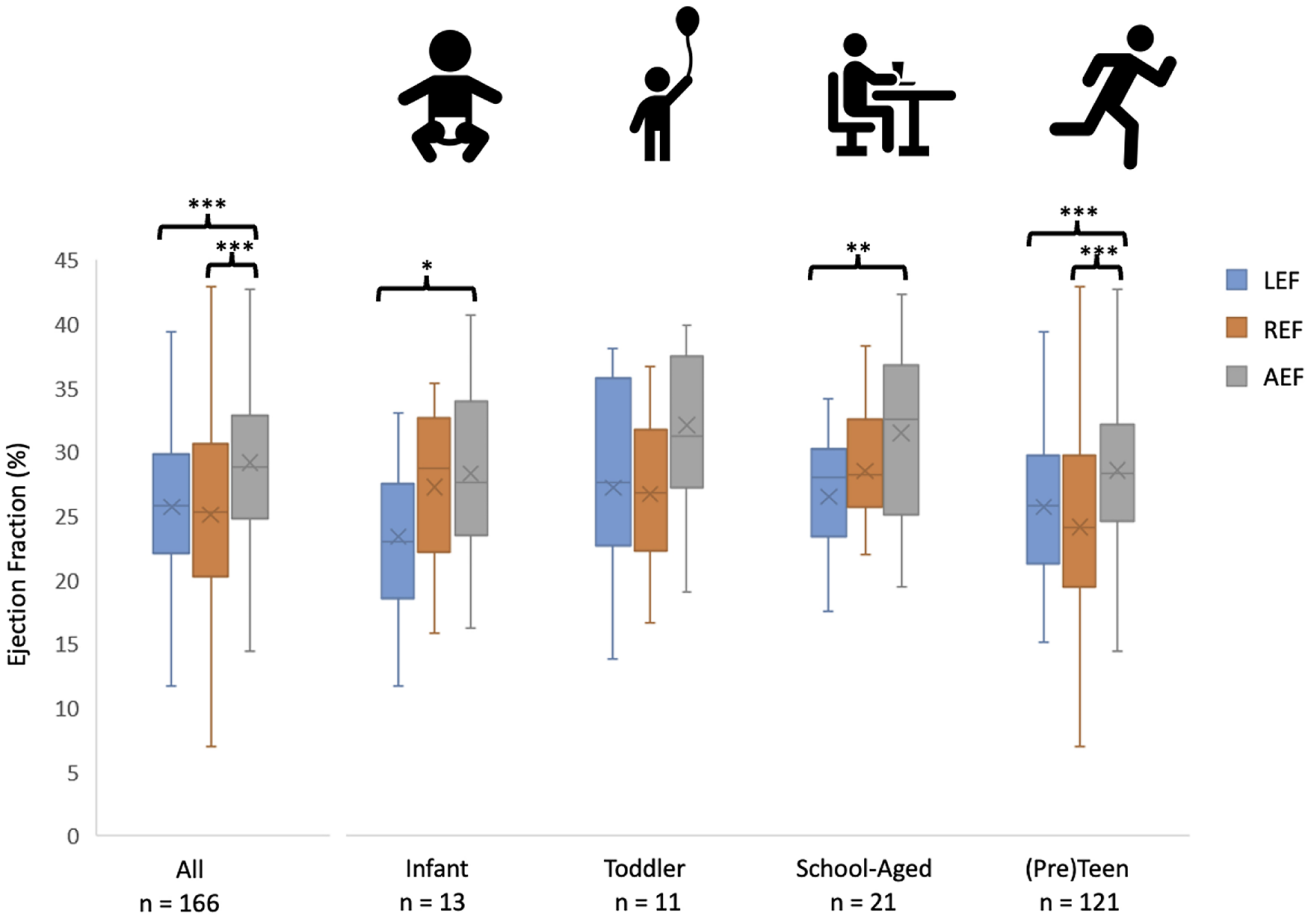
A comparison of the contributions of longitudinal, radial and anteroposterior contraction to global RV function. Individual and mean values of LEF, REF, and AEF are shown in the entire population (leftmost column) as well as the different age categories, with statistical comparison among the three motion components. **p < 0.01, ***p < 0.001.

Figure 2 shows the ejection fraction components for the entire cohort as well as broken down by age group. For the entire cohort, the AEF was greater than the other two components; the same pattern was observed for the oldest group. For the school-aged cohort, the AEF was greater than the LEF. No significant differences were observed among any components in the infants and toddlers.

There was excellent inter-center reproducibility with intraclass correlation coefficients of 0.97 (95% CI 0.94–0.98) for RV end-diastolic volume and 0.94 (95% CI 0.89–0.97) for RV end-systolic volume. Bland Altman plots are presented as Supplementary Figure S1. As the ReVISION method is a fully automated technique, it adds no further variability in addition to that represented in these volume comparisons.

## Discussion

The primary aim of this two-center study was to define the specific contributions of longitudinal, radial and anteroposterior contraction to global RV function in a cohort of healthy children using 3D echocardiographic images and advanced analytical software. As well, we sought to describe the maturational changes that occur in each of the components of RV function, in addition to the global value. Our major finding was that whereas the contributions of the longitudinal and radial components were similar, a predominance for AP contraction was present in the overall cohort. Moreover, age-related differences were present for global RVEF, REF, REFi, and all compenents fof RV strain.

There are three primary mechanisms of RV contraction: longitudinal shortening with traction of the tricuspid valve annulus toward the apex, inward (radial) movement of the free wall, and anteroposterior directed motion of the RV wall related to LV deformation (15, 21). In this study, we identified the predominance of anteroposterior shortening in nearly every age group. Typical 2D parameters of RV function have relied upon simple, linear measurements which incompletely reflect the complex mechanics of RV function. For example, TAPSE is a measure of the longitudinal motion of the chamber, has been shown to correlate with global RVEF, and has been described in children with pulmonary hypertension with and without congenital heart disease (22). FAC is a measurement of both the radial contraction of the free wall as well as longitudinal traction of the tricuspid annulus and has been associated with changes in RV function in patients with Ebstein anomaly undergoing the cone procedure (23). While these techniques offer some degree of quantitative analysis of RV function, they are imperfect in that they do not account for RV function in all axes, failing to fully quantify the complex RV mechanics. Moreover, they do not meaningfully assess the anteroposterior contraction, which predominated in our study.

Prior groups have used 2D echocardiography to show that the contraction pattern of the RV in children changes over the first year of life as children transition from fetal circulation in which the RV faces a high afterload to post-natal circulation in which the pulmonary vascular resistance gradually declines over the first few months of life. One group used measurements of TAPSE and a surrogate marker of radial contraction to demonstrate a clear transition from predominantly radial contraction to more longitudinal contraction around 4 months of age (24). While likely underpowered to detect significant differences between the contribution of radial contraction in neonates (i.e., under 1 month of age) and older children, we did find differences in the contribution of radial shortening with increasing age. Others have used 2D speckle-tracking strain analysis to demonstrate that longitudinal contraction increases in this first year of life in premature infants (24), whereas our study suggests that pattern of longitudinal shortening may be complex, with an early increase in magnitude followed by a later return to baseline values. Moreover, we are the first to describe anteroposterior contraction patterns in children. The anteroposterior contraction is determined in large part by the circumferential shortening of the LV mid-layer myofibers, which draw the RV free-wall insertion lines towards each other. It has been shown previously that AEF is strongly associated with LVEF in both healthy volunteers and those with congenital heart disease resulting in a systemic RV (25, 26).

The maturational differences in directional contraction of the RV identified in this work emphasize the importance of using advanced techniques to assess RV contraction patterns in children with simple and complex congenital heart disease. Prior studies have demonstrated the prognostic value of global RV function in children with congenital heart disease (27, 28). Even in the face of preserved global RV function, important variations in the relative contributions of the three main components can be seen, as in the case of adults undergoing mitral valve surgery as well as those with either volume-loading or pressure-loading lesions on the right side of the heart (25, 29, 30). Understanding and quantifying the relative contribution of each component of RV contraction could have potential applications across multiple subsets of RV pathology by providing insights into the long-term effects of (1) pressure-load on the RV in children with chronic RV outflow tract obstruction (i.e., children with tetralogy of Fallot, congenital pulmonic stenosis, idiopathic pHTN), (2) volume-load in children with long-standing left-right shunt (i.e., partial anomalous pulmonary venous connections, atrial septal defects), (3) primary RV myopathy (i.e., arrhythmogenic cardiomyopathy) and finally (4) complex anatomy leading to the use of the right ventricle as the systemic ventricle in either a single ventricle circulation (i.e., hypoplastic left heart syndrome) or a biventricular circulation (i.e., “congenitally corrected” transposition of the great arteries). A more refined understanding of the evolution and progression of changes in RV contraction could help providers identify the effects of medical therapy and better define the optimal timing for procedural interventions.

## Limitations

Our study group was limited by smaller number of subjects in the younger age groups, which is of particular importance in considering the significant hemodynamic changes to which the RV is subjected in the first weeks to months of life. Moreover, the differing recruitment methods contributed to a predominance of males in the oldest age group, with all but 9 subjects at the Semmelweiss site being male. As a consequence, our exploratory analysis intended to identify sex-specific differences in ejection fraction parameters among various age groups was likely underpowered. Apart from the limitations to the study population itself, there is no “gold standard” for comparison of our specific results because there is, at present, no reference method for assessing the relative components of RV motion.

## Conclusions

In healthy children, analysis of the components of right ventricular contraction is feasible and reliable. In this pediatric cohort, the anteroposterior component of RV contraction was greater than the radial and longitudinal contributions. Additionally, there were age-related differences for both global RVEF and the radial component of RV contraction. Future 3DE-based study of the contraction patterns of the pediatric right ventricle, especially in children with congenital heart disease, may facilitate enhanced recognition of dysfunction and assessment of treatment effects.

## Data Availability

The raw data supporting the conclusions of this article will be made available by the authors, without undue reservation.
