# Supplementary figures and images for: Right ventricular contraction patterns in healthy children using three-dimensional echocardiography
Source: Front Cardiovasc Med. 2023 Aug 3;10:1141027. doi: 10.3389/fcvm.2023.1141027 (PMC10435279; doi:10.3389/fcvm.2023.1141027)

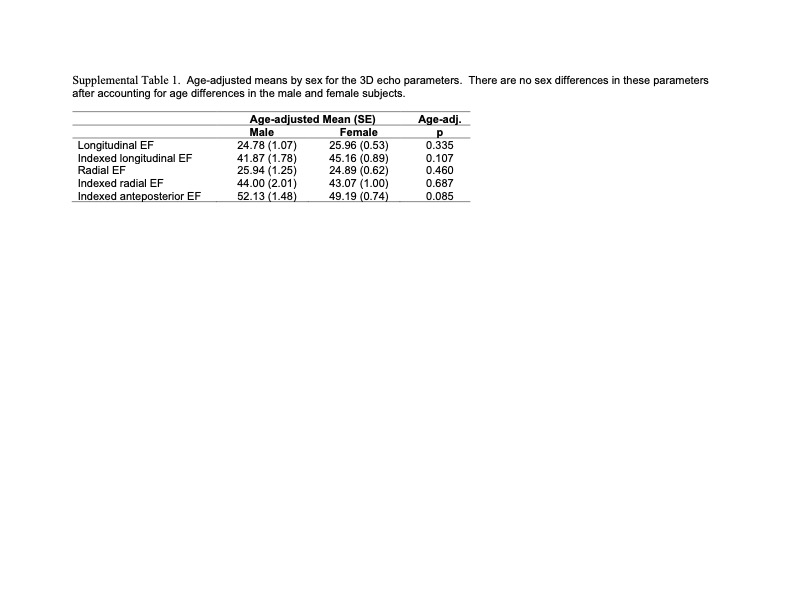

Supplement: Supplementary file 1 [file Image1.jpeg]

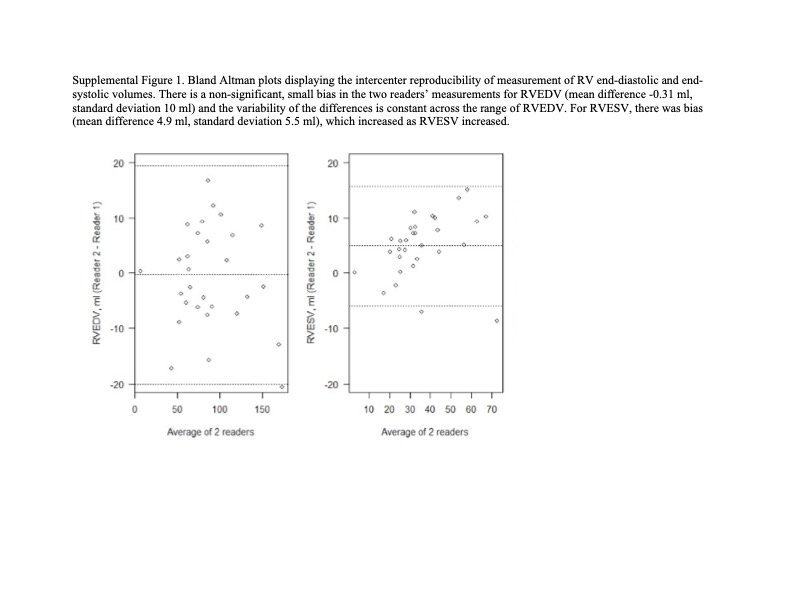

Supplement: Supplementary file 2 [file Image2.jpeg]
